# A case of dimethyl sulfoxide-induced seizure in multiple myeloma

**DOI:** 10.1007/s44313-025-00063-9

**Published:** 2025-03-04

**Authors:** Ho Cheol Jang, Ga-Young Song, Mihee Kim, Seo-Yeon Ahn, Jae-Sook Ahn, Deok-Hwan Yang, Hyeoung-Joon Kim, Je-Jung Lee, Sung-Hoon Jung

**Affiliations:** Department of Hematology-Oncology, Chonnam National University Hwasun Hospital, 322 Seoyangro, Hwasun, Jeollanamdo 58128 Republic of Korea

## To the Editor

Multiple myeloma (MM) is a malignant plasma cell disorder characterized by the clonal proliferation of plasma cells in the bone marrow, leading to extensive skeletal destruction, renal failure, anemia, and hypercalcemia. It is the second most common hematologic malignancy, accounting for approximately 10% of hematologic cancers and 1% of all cancers [1, 2]. Over the past few decades, advancements in MM treatment have significantly improved patient outcomes, incorporating therapies such as proteasome inhibitors, immunomodulatory drugs, monoclonal antibodies, and autologous stem cell transplantation (ASCT) [3, 4]. ASCT remains a key therapeutic strategy, offering improved survival rates and prolonged remission for eligible patients [4]. The increasing adoption of ASCT in MM is supported by recent data highlighting its benefits [5].

During ASCT, peripheral blood stem cells are collected from the patient through peripheral blood stem cell collection (PBSCC) and cryopreserved for future use. Dimethyl sulfoxide (DMSO) is commonly used as a cryoprotectant to prevent cell damage during freezing and thawing [6]. While DMSO is effective and generally safe, it has been associated with various side effects, including nausea, vomiting, and, in rare cases, seizures [7, 8]. Seizures induced by DMSO during stem cell infusion are uncommon but have been documented in the literature, emphasizing the need for awareness and prompt management during the transplantation process [9].

Here, we present the case of a 55-year-old female with MM who experienced a seizure during ASCT, detailing the clinical course, management strategies, and implications for future practice.

### Case

A 55-year-old female with MM underwent treatment with six cycles of an revlimid, Velcade, and dexamethasone. (RVd) regimen, consisting of bortezomib, dexamethasone, and lenalidomide. After three cycles of RVd, PBSCC was performed over two consecutive days, yielding a total of 11 × 10^6^ CD34^+^ cells/kg. The collected stem cells were cryopreserved in seven separate bags. The patient received a conditioning regimen of high-dose melphalan for two days (Day −4 and Day −3), followed by a two-day rest period (Day −2 and Day −1), with ASCT scheduled on Day 0.

On the day of transplantation, the cryopreserved stem cells, treated with DMSO, were thawed and infused. The patient tolerated the infusion well until the administration of the fifth bag. During the infusion of the sixth bag, the patient became drowsy but remained responsive to verbal stimuli. The infusion rate was reduced, and the administration continued. However, during the infusion of the seventh bag, the patient developed snoring respirations and became unresponsive to painful stimuli. She exhibited whole-body rigidity, experienced urinary incontinence, and vomited once. These symptoms were accompanied by a generalized tonic-clonic seizure lasting approximately two minutes.

**Fig. 1:**
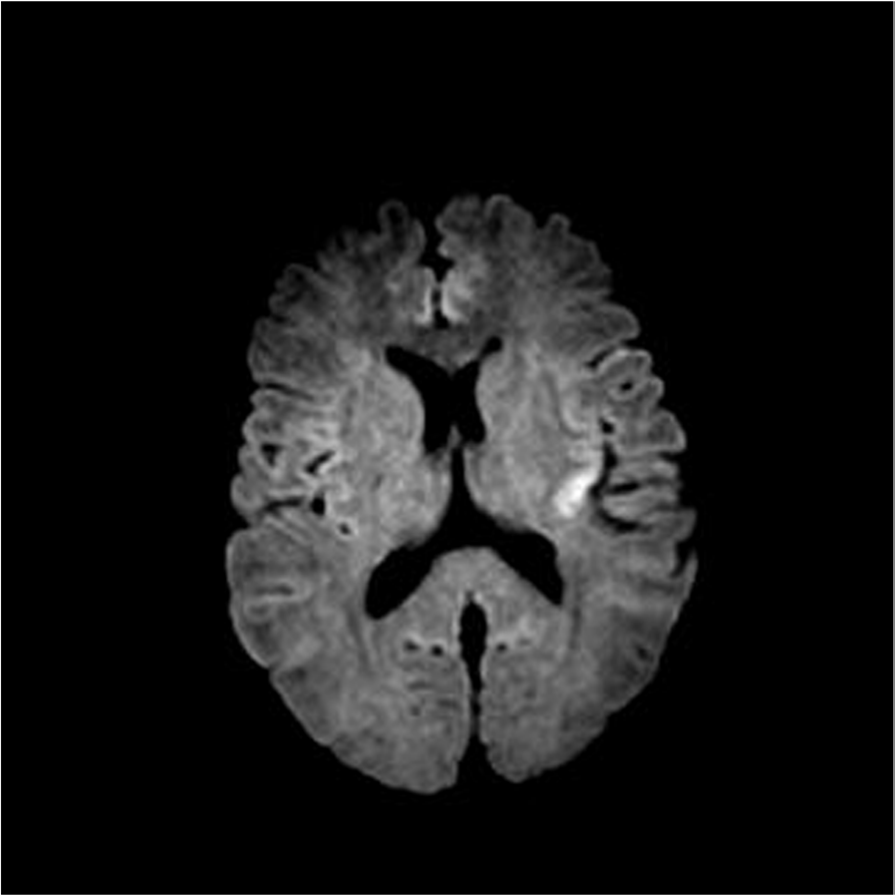
Magnetic resonance imaging of the brain demonstrated focal diffusion-restricted lesions in the left insular lobe and left centrum semiovale, suggesting seizure-related changes rather than acute infarction

Immediate management included the administration of lorazepam (2 mg) and levetiracetam (500 mg), which successfully controlled the seizure activity. A comprehensive evaluation was promptly initiated. Laboratory tests, including a complete blood count, electrolyte panel, and renal function tests, revealed no significant abnormalities (Table 1). A non-contrast computed tomography scan of the brain showed no evidence of acute large-vessel infarction, hemorrhage, or hydrocephalus. Additionally, a subsequent magnetic resonance imaging scan was consistent with an acute seizure event rather than acute infarction (Fig. 1). Consequently, an electroencephalogram could not be performed due to the patient’s isolation in a sterile room. No additional seizures were observed during the isolation period.

The patient had received melphalan as part of the conditioning regimen two days before ASCT, followed by a two-day rest period before stem cell infusion on Day 0. At the time of the seizure, no antibiotics, including cefepime, were being administered. The absence of electrolyte imbalances, combined with the temporal correlation between the seizure event and the infusion of DMSO-containing stem cells, suggested that DMSO toxicity was the most likely etiology. Following the seizure, the patient was monitored closely, with no further seizure activity observed. She remained stable and showed signs of successful engraftment in the following weeks, as evidenced by increasing neutrophil and platelet counts. The patient was discharged in good condition and scheduled for regular outpatient follow-up to monitor her post-transplant recovery and hematologic response.

## Discussion

This case highlights a seizure event induced by DMSO during ASCT in a patient with MM. DMSO is widely used as a cryoprotectant in hematopoietic stem cell transplantation to preserve cell viability during freezing and thawing processes [8].

Our patient experienced a generalized tonic-clonic seizure following the infusion of a cryopreserved stem cell bag containing DMSO. The strong temporal relationship between DMSO administration and the onset of neurological symptoms suggests that DMSO toxicity was the most likely etiology. Although the precise mechanism by which DMSO induces seizures is not fully understood, it is hypothesized that DMSO may alter neuronal membrane stability or interfere with neurotransmitter function, leading to seizure activity.

ASCT is a standard treatment modality for patients with MM, and the increasing use of this approach emphasizes the need to recognize and manage rare but clinically significant complications such as DMSO-induced seizures. Several case reports have underscored the necessity for heightened awareness and preparedness regarding potential adverse effects associated with DMSO infusion. Further investigation is required to clarify the underlying pathophysiology of DMSO-induced seizures and to identify clinical factors that may predict patients at higher risk. Currently, there are no established clinical predictors for DMSO-induced seizures. However, one study suggested that DMSO toxicity exhibits dose-dependent characteristics. Strategies aimed at reducing the total daily dose of DMSO, such as dividing the infusion over two consecutive days, have been proposed as potential methods to mitigate seizure risk [10]. Additional studies, including multicenter analyses and retrospective evaluations of patients who develop DMSO-induced seizures, are warranted to improve risk stratification and preventative measures.

**Table 1:** Laboratory test results on the morning of ASCT, at the time of the seizure episode, and on the morning of the following day after ASCT

Variable	Before ASCT	At the time point of seizure	Day 1 after ASCT
WBC count (× 10^3^/μL)	1.9	7.6	5.9
Hgb (g/dL)	9.9	10.1	9.0
Platelet count (× 10^3^/μL)	146	164	94
Lymphocyte (× 10^3^/μL)	0.02	0.89	0.05
Monocyte (× 10^3^/μL)	0.01	0.14	0.01
Neutrophil (× 10^3^/μL)	1.85	6.51	5.84
Eosinophil (× 10^3^/μL)	0.01	0.01	0
Basophil (× 10^3^/μL)	0.01	0.06	0.01
Total calcium (mg/dL)	8.3	8.8	8.1
Magnesium (mg/dL)	2.08	2.12	2.01
Inorganic phosphorus (mg/dL)	3.1	2.5	2.8
Sodium (mEq/L)	138	140	142
Potassium (mEq/L)	3.8	3.8	3.4
Chloride (mEq/L)	107	107	109
AST (U/L)	26	173	88
ALT (U/L)	20	33	33
Total bilirubin (mg/dL)	1.03	1.02	0.88
BUN (mg/dL)	12.5	13.2	14.4
Creatinine (mg/dL)	0.35	0.56	0.39
CRP (mg/dL)	0.20	0.34	1.01

*Abbreviations*: *ASCT* autologous stem cell transplantation, *WBC* white blood cell, *Hgb* hemoglobin, *AST* aspartate aminotransferase, *ALT* alanine aminotransferase, *BUN* blood urea nitrogin, *CRP* C-reactive protein

## Data Availability

No datasets were generated or analysed during the current study.
